# Substantial improvement in short-term survivorship in patients receiving robotic arm-assisted medial unicompartmental knee arthroplasty: a retrospective cohort study

**DOI:** 10.1302/2633-1462.710.BJO-2026-0104.R1

**Published:** 2026-10-13

**Authors:** Tim Cheok, Kristopher Law, Julie F. Vermeir, Ruurd L. Jaarsma, Job N. Doornberg, Anthony M. Silva

**Affiliations:** 1 Department of Orthopaedic Surgery, The Prince Charles Hospital, Brisbane, Australia; 2 College of Medicine and Public Health, Flinders University, Adelaide, Australia; 3 Graduate School of Medical Sciences, University of Groningen, Groningen, The Netherlands; 4 Department of Orthopaedic Surgery, St Vincent’s Private Hospital Northside, Brisbane, Australia; 5 Faculty of Health, Medicine and Behavioural Sciences, The University of Queensland, Brisbane, Australia; 6 Faculty of Health, Queensland University of Technology, Brisbane, Australia; 7 Department of Orthopaedic Surgery, Flinders Medical Centre, Adelaide, Australia; 8 Department of Orthopaedic Surgery, University Medical Center Groningen, Groningen, The Netherlands; 9 Department of Orthopaedic Surgery, The Wesley Hospital, Brisbane, Australia

**Keywords:** Unicompartmental Knee, Robotic, Mako, Conventional, Survivorship, Instrumented, robotic arm, Unicompartmental knee arthroplasty (UKA), surgical site infection, Anesthesiologists, osteoarthritis, primary surgeon, BMI, Charlson Comorbidity Index, unicompartmental knee, medial unicompartmental knee arthroplasty

## Abstract

**Aims:**

Robotic arm-assisted unicompartmental knee arthroplasty (R-UKA) has been shown to improve alignment and accuracy of component placement compared with conventional unicompartmental knee arthroplasty (C-UKA). Despite this, there is conflicting evidence for whether this has an impact on revision rates.

**Methods:**

We performed a retrospective analysis of a prospective database, comparing patients who underwent R-UKA using the Stryker Restoris MCK implant with those who underwent C-UKA using the cemented Oxford Partial Knee implant, between January 2002 and December 2024 at The Prince Charles Hospital (Brisbane, Australia), with a minimum six-month follow-up. Patient selection for UKA mirrored the recommendations by Kozinn and Scott. The primary outcome of interest was to compare the all-cause revision rate between these two groups. Secondary outcomes of interest were to compare hospital length of stay (HLOS), surgical duration, the odds of superficial surgical site infection, and the odds of periprosthetic joint infection (PJI) between the same groups. The analysis of individual outcomes were adjusted for age, sex, BMI, American Society of Anesthesiologists grade, Charlson Comorbidity Index, and primary surgeon grade.

**Results:**

The hazard of all-cause revision significantly decreased by 78% in the R-UKA group as compared with the C-UKA group (p = 0.046). HLOS was also significantly shorter by 2.2 days (95% CI 1.0 to 3.3) in the R-UKA group (p < 0.001). There was no significant difference in the surgical duration (p = 0.733), odds of superficial surgical site infection (p = 0.845), nor in the odds of PJI (p = 0.415) between the two groups.

**Conclusion:**

The substantial improvement in revision outcomes suggests that R-UKA is the option of choice for managing symptomatic unicompartmental knee arthritis in carefully selected patients with isolated medial compartmental osteoarthritis, especially in the hands of low-volume surgeons. Decreased HLOS should be interpreted with caution, given the non-contemporaneous nature of the two cohorts.

Cite this article: *Bone Jt Open* 2026;7(10):1304–1311.

## Introduction

Unicompartmental knee arthroplasty (UKA) is an established surgical method to address symptomatic isolated unicompartmental knee arthritis in select patients.^1^ As compared with a total knee arthroplasty, UKA preserves normal joint kinematics, thus leading to a faster recovery and improved patient-reported outcomes.^2-5^ Despite these benefits, UKA may be underutilized due to the high technical demands of the surgery.^6^ Conventional instrumented UKA (C-UKA) has a steep learning curve of between 25 and 50 cases,^7-9^ with further studies showing inferior outcomes when performed by low-volume surgeons.^10-12^

Although the most common indication for revision following UKA is for progression of arthritis, early failures have been attributed to technical errors in achieving adequate implant positioning.^13,14^ Malalignment has been shown to cause accelerated polyethylene wear, early aseptic loosening, stress fractures of the adjacent compartment, worse functional outcomes, and ultimately expedite the progression of osteoarthritis.^15-20^ In order to mitigate this risk, robotic arm-assisted UKA (R-UKA) systems with haptic feedback technology have been developed to improve the accuracy of bony resections and enhance implant positioning.^21,22^ Of concern, a previous study utilizing data from the Australian Orthopaedic Association National Joint Replacement Registry (AOANJRR) has reported a fivefold increase in the revision rate for periprosthetic joint infection (PJI) in patients undergoing the Mako-assisted Restoris joint arthroplasty (Stryker, USA), compared with other non-robotically assisted UKA in the first three months.^23^ This finding has now not been shown in the 2025 report.^24^

Several studies comparing R-UKA with C-UKA have shown improved functional outcomes and shorter length of stay in hospital. Of interest, these studies had reported conflicting results on the effect of R-UKA on complication rates.^25-27^ Thus, the primary aim of this study was to compare the all-cause revision rate between patients undergoing R-UKA versus those undergoing C-UKA at our institution. Secondary outcomes of interest were to compare hospital length of stay (HLOS), surgical duration, the odds of superficial surgical site infection, and the odds of PJI between these two groups.

## Methods

This retrospective cohort study was conducted at The Prince Charles Hospital, a tertiary-level university teaching hospital located in Brisbane, Australia. No AI was used in the research or manuscript development,^28^ and reporting of this study adhered to the recommendations by STROBE.^29^ We used prospectively collected data from our institution’s internal audit database to conduct this study, thereby minimizing the risk of recall bias. In selected patients, UKA has been the standard of care for symptomatic unicompartmental knee arthritis at our institution. Patient eligibility criteria for UKA at our institution closely mirrored those of Kozinn and Scott:^30^ unicompartmental knee arthritis, non-manual occupation, range of motion ≥ 90°, flexion contracture ≤ 5°, correctable valgus/varus deformity of < 15°, as well as the absence of inflammatory arthritis. Prior to 2019, only C-UKAs were performed at our institution, utilizing a variety of implants. Since then, there has been a departmental shift in practice towards the use of R-UKA.

Consecutive patients who underwent an elective UKA at The Prince Charles Hospital between January 2002 and December 2024, with a minimum follow-up period of six months, were identified for potential inclusion. All patients who underwent R-UKA received a Restoris MCK Partial Knee (Stryker, USA). This is a cemented fixed-bearing prosthesis implanted with the assistance of the Mako robotic system (Stryker), an image-guided semiactive robotic arm technology with haptic feedback. As the majority of patients who underwent a C-UKA received the cemented Oxford Partial Knee (Zimmer Biomet, USA), we used this as our comparator group to minimize the risk of anomalous outcomes from implant heterogeneity. This is a cemented mobile bearing prosthesis, which has been implanted using the instrumented technique as described by the manufacturer. We excluded patients who underwent lateral compartment joint arthroplasty, those who received a bilateral simultaneous UKA, and those who underwent C-UKA utilizing implants apart from the Oxford Partial Knee System. All UKAs were performed by or under the supervision of a fellowship-trained consultant orthopaedic surgeon (AMS).

Using our predefined inclusion and exclusion criteria, we identified 177 patients for analysis: 65 patients in the C-UKA group and 112 patients in the R-UKA group. The median follow-up was 11.8 years (IQR 7.7 to 20.0) in the C-UKA group and 4.6 years (IQR 2.6 to 5.5) in the R-UKA group. There was no significant difference in age at the time of surgery (p = 0.211, Mann-Whitney U test), sex (p = 0.158, Fisher’s exact test), BMI (p = 0.209, Mann-Whitney U test), American Society of Anesthesiologists (ASA)^31^ grade (p = 0.322, Fisher’s exact test), smoking status (p = 0.535, Fisher’s exact test), Charlson Comorbidity Index (CCI)^32^ (p = 0.893, Mann-Whitney U test), or primary surgeon grade (p = 0.618, Fisher’s exact test) between the two groups. In terms of comorbidities, there were a significantly greater number of patients with chronic obstructive pulmonary disease (p = 0.035, Fisher’s exact test) and depression/anxiety (p = 0.011, Fisher’s exact test) in the R-UKA group compared with the C-UKA group. Significantly fewer patients in the R-UKA group had a previous meniscectomy (p = 0.048, Fisher’s exact test) compared with the C-UKA group. There was no difference in the proportion of patients with a history of cardiac arrhythmia (p = 0.539, Fisher’s exact test), coronary artery disease (p > 0.999, Fisher’s exact test), cardiac failure (p = 0.134, Fisher’s exact test), cerebrovascular disease (p > 0.999, Fisher’s exact test), chronic kidney disease (p = 0.501, Fisher’s exact test), diabetes (p = 0.194, Fisher’s exact test), hypertension (p = 0.210, Fisher’s exact test), chronic liver disease (p > 0.999, Fisher’s exact test), and peripheral vascular disease (p = 0.533, Fisher’s exact test) between the two groups. In both groups, the consultant surgeon was the primary operator in most cases. A detailed analysis of these covariates is displayed in Table I.

**Table I.** Demographic details, comorbidities, and surgical factors.

Variable	C-UKA	R-UKA	p-value
Patients, n	65	112	
Median age at surgery, yrs (IQR)	64.0 (56.0 to 73.0)	66.0 (59.0 to 72.5)	0.211*
**Sex, n (%)**			0.158†
Male	31 (47.7)	67 (59.8)	
Female	34 (52.3)	45 (40.2)	
Median BMI, kg/m² (IQR)	29.6 (26.8 to 38)	28.4 (26.1 to 32.1)	0.209*
**ASA grade, n (%)**			0.322†
I	10 (15.4)	11 (9.8)	
II	35 (53.9)	53 (47.3)	
III	20 (30.8)	46 (41.1)	
IV	0 (0)	2 (1.8)	
**Comorbidities, n (%)**			
Cardiac arrhythmia	3 (4.6)	9 (8.0)	0.539†
Coronary artery disease	6 (9.2)	10 (8.9)	> 0.999†
Cardiac failure	2 (3.1)	0 (0)	0.134†
Chronic obstructive pulmonary disease	3 (4.6)	16 (14.3)	0.035†
Cerebrovascular disease	2 (3.1)	3 (2.7)	> 0.999†
Chronic kidney disease	5 (7.7)	5 (4.5)	0.501†
Depression/anxiety	3 (4.6)	21 (18.8)	0.011†
Diabetes	12 (18.5)	14 (12.5)	0.194†
Hypertension	25 (38.5)	55 (49.1)	0.210†
Chronic liver disease	1 (1.5)	1 (0.9)	> 0.999†
Peripheral vascular disease	0 (0)	2 (1.8)	0.533†
Previous meniscectomy	3 (4.6)	0 (0)	0.048†
Active smoker	5 (7.7)	6 (5.4)	0.535†
Charlson Comorbidity Index score	2.0 (1.0 to 4.0)	2.0 (2.0–4.0)	0.893†
**Primary surgeon grade, n (%)**			0.699†
Consultant	38 (58.5)	72 (64.3)	
Fellow	13 (20.0)	18 (16.1)	
Resident	14 (21.5)	22 (19.6)	

* Mann–Whitney U test

† Fisher’s exact test

ASA, American Society of Anesthesiologists; C-UKA, conventional unicompartmental knee arthroplasty; R-UKA, robotic arm-assisted unicompartmental knee arthroplasty.

### Outcome measurement

The primary outcome of this study was to compare the all-cause revision rate between the R-UKA and C-UKA groups. To account for variation in revision rates due to the differences in follow-up period, we employed survivor analysis, and examined revision incidence within three years following UKA. Survival time was defined as the interval from the date of UKA to the date of revision surgery. Patients were censored at death or in the case of survival, on 1 June 2025. Kaplan-Meier analysis was used to illustrate the differences between the two arms. This was quantified using hazard ratios (HRs) derived from a Cox-regression analysis. Furthermore, the difference in revision incidence within five years of UKA was quantified using odds ratios (ORs).

The secondary outcomes were to compare the median HLOS and surgical time, as well as the odds of superficial surgical site infection and PJI between the two groups. To further account for difference in patient factors and surgeon experience, each outcome was adjusted for age, sex, BMI, ASA grade, CCI score and primary surgeon grade using the appropriate regression analyses.

### Statistical analysis

Continuous variables were expressed as median (IQR) and compared using Mann-Whitney U test, whereas discrete variables were expressed as count (percentage) and compared using Fisher’s exact test. All statistical analyses were performed using Stata v. 18.0 (StataCorp, USA). The threshold for statistical significance was set at p < 0.05.

## Results

A total of 15 revisions were identified within the follow-up period: 12 (18.5%) in the C-UKA group and three (2.7%) in the R-UKA group. In the C-UKA group, four patients underwent revision due to progression of osteoarthritis, another four patients were revised for aseptic loosening, and one patient each revised for bearing dislocation, postoperative periprosthetic fracture, malalignment, and PJI. Of the three patients that were revised in the R-UKA group, two were for aseptic loosening and one for progression of arthritis. Median time to revision was 3.3 years (IQR 0.8 to 14.1) in the C-UKA group and 1.9 years (IQR 0.9 to 3.1) in the R-UKA group. The Kaplan-Meier survival curve is illustrated in Figure 1. After adjusting for age, sex, BMI, ASA grade, CCI score, and primary surgeon grade, the all-cause revision rate was significantly lower in the R-UKA group (HR 0.22, 95% CI 0.05 to 0.98; p = 0.046, Wald test). Seven patients were revised within three years of their index operation: five (7.7%) in the C-UKA group and two (1.8%) in the R-UKA group. After adjusting for age, sex, BMI, ASA grade, CCI score, and primary surgeon grade, the revision hazard was 99% lower in the R-UKA group (p = 0.023; Wald test). A detailed breakdown of the timeline for each revision complication is discussed in Supplementary Table i.

**Fig. 1:**
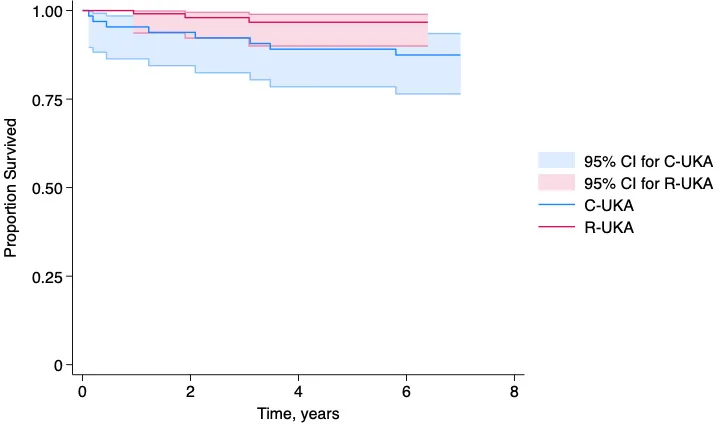
Kaplan-Meier survival analysis truncated at seven years. C-UKA, conventional unicompartmental knee arthroplasty; R-UKA, robotic arm-assisted unicompartmental knee arthroplasty.

Median HLOS was five days (IQR 3.0 to 6.0) in the C-UKA group and three days (IQR 2.0 to 3.5) in the R-UKA group. When adjusted for age, sex, BMI, ASA grade, CCI score, and primary surgeon grade, the HLOS was significantly lower by 2.2 days (95% CI 1.0 to 3.3) in the R-UKA group (p < 0.001, Wald test) when compared with the C-UKA group. Similarly, median surgical time was 105.0 minutes (IQR 103.0 to 126.0) and 98.0 minutes (IQR 98.0 to 123.0), respectively. Adjusted difference in surgical time was three minutes shorter (95% CI -15.35 to 21.41) in the R-UKA group. This was not statistically significant (p = 0.733; Wald test). Two infections were recorded: one superficial surgical site infection in the R-UKA group and one PJI in the C-UKA group. The adjusted odds of superficial surgical site infection was 0.73 (95% CI 0.03 to 16.96; p = 0.845, Wald test), and the adjusted odds of PJI was 0.30 (95% CI 0.01 to 12.76; p = 0.415, Wald test) in the R-UKA group, compared with the C-UKA group. The outcomes of our analysis are summarized in Table II.

**Table II.** Summary of primary and secondary outcomes.

Variable	C-UKA	R-UKA	Adjusted analysis (95% CI)	p-value
Patients, n	65	112		
**All-cause revision, n (%)**	12 (18.5)	3 (2.7)	HR 0.22 (0.05 to 0.98)	0.046*
Aseptic loosening	4	2		
Bearing dislocation	1	0		
Periprosthetic fracture	1	0		
Malalignment	1	0		
Periprosthetic joint infection	1	0		
Progression of arthritis	4	1		
**Three-year revision, n (%)**	5 (7.7)	2 (1.8)	HR 0.01 (0.00 to 0.54)	0.023*
Aseptic loosening	1	2		
Periprosthetic fracture	1	0		
Malalignment	1	0		
Periprosthetic joint infection	1	0		
Progression of arthritis	1	0		
Median hospital length of stay, days (IQR)	5.0 (3.0 to 6.0)	3.0 (3.0 to 3.5)	*β* -2.2 (-3.3 to -1.0)	< 0.001†
Median surgical duration, minutes (IQR)	105.0 (103.0 to 126.0)	98.0 (86.0 to 123.0)	*β* -3.0 (-21.4 to 15.3)	0.733†
Superficial surgical site infection, n (%)	0 (0)	1 (0.89)	OR 0.73 (0.03 to 17.0)	0.845*
Periprosthetic joint infection, n (%)	1 (1.5)	0 (0)	OR 0.30 (0.02 to 5.5)	0.415*

* Fisher’s exact test

† Mann–Whitney U test

C-UKA, conventional unicompartmental knee arthroplasty; HR, hazard ratio; OR, odds ratio; R-UKA, robotic arm assisted unicompartmental knee arthroplasty; β, regression coefficient.

## Discussion

The most pertinent finding of this study is that patients undergoing R-UKA at our institution had a significantly lower rate of all-cause revision compared with those undergoing C-UKA. Our results echo the most recent AOANJRR report, which has found a 36% reduction in revision hazard in R-UKA compared with C-UKA after 3.5 years.^24^ As the assumption of proportionality was upheld in our analysis, we did not show a divergent pattern of survivorship as reported in the AOANJRR. Unique to the present study is that we demonstrated a significant reduction in revision rate following medial R-UKA. Several previous studies have only shown a reduction in complication rate, with no effect on revision rate itself.^25-27,33,34^ We postulate that this finding may be due to optimization of component positioning. Our hypothesis is supported by several high-quality randomized controlled trials that have proven that R-UKA significantly improves the accuracy of component placement,^22,35^ with additional studies showing that this benefit was sustained even in inexperienced surgeons.^36,37^ The results of our primary outcome analysis thus support the adoption of robotic technology in UKA to improve patient outcome.

A previous AOANJRR study by St Mart et al^23^ reported an increase in the revision rate for PJI in patients undergoing the Mako-assisted Restoris joint arthroplasty in the first three months. This difference was hypothesized to result from the additional wounds caused by the introduction of guide pins. Fortunately, we did not observe any increase in infection rate within our patient population. Only one patient in the C-UKA group was revised due to infection. This patient received a washout and debridement with exchange of the polyethylene liner, followed by a period of antibiotic treatment. Additionally, one patient in the R-UKA group also had a superficial surgical site infection unrelated to the pin sites. This patient was treated with a short course of antibiotics. Both of these patients were closely followed up in an outpatient setting, with no evidence of recurrence of infection. We also demonstrated a significantly lower median HLOS in the R-UKA group by two days. This finding has also been reported by several other studies, and is likely secondary to improved pain scores from less traumatic soft-tissue handling.^21,25,38-40^ In contrary to the findings by Goh et al,^41^ there was no statistically significant difference in surgical duration between the two groups. Therefore, despite a higher consumable cost associated with R-UKA, the decrease in revision rate and shorter HLOS have been shown to provide overall savings for the health system.^42^

Our study is the first to report a substantial reduction in all-cause revision for patients undergoing medial R-UKA compared with those undergoing medial C-UKA. A major strength of this study is the use of a prospectively maintained database, thus minimizing the risk of recall bias. We also accounted for differences in patient factors and surgeon experience by adjusting each outcome for age, sex, BMI, ASA grade, CCI score, and primary surgeon grade. Although no a priori power analysis was performed given the retrospective nature of this study, post-hoc power analysis showed that our study is adequately powered to detect the primary outcome. There are several weaknesses of this study that need to be addressed. First, the follow-up period was unequal between the two arms, with C-UKA having a longer follow-up period. We mitigated this difference by the use of survival analysis, which takes into consideration follow-up duration, as well as a truncated survivorship model.^43^ In our institution, no patient received a C-UKA after 2019, making contemporaneous cohort examination unfeasible. Second, the implant used in the R-TKA arm was a fixed bearing prosthesis, whereas the implant used in the C-UKA was a mobile bearing implant. Several registry studies have failed to demonstrate a difference in long-term outcome attributed to implant design.^44,45^ Third, patient-reported outcome measures (PROMs) were not collected in our cohort, limiting the inferences that could be derived out of this study. Nonetheless, several studies have consistently demonstrated improved short-term functional outcomes following R-UKA,^27,34,46^ further reinforcing the benefits of this technological advancement. Fourth, it is important to note that there was a difference in times between R-UKA and C-UKA groups. Evolution of enhanced recovery protocols, as well as changing perceptions on early hospital discharge over time makes HLOS data less reliable. Lastly, we did not assess whether the poorer outcomes associated with C-UKA at our institution were attributable to the associated learning curve, and thus not generalizable to high-volume surgeons or centres. Approximately 40% of cases were performed by a fellow or resident physician in both groups. It is entirely possible that the outcomes may be equivalent if the operating surgeon had overcome the steep learning curve associated with C-UKA. Previous studies have found that the Mako-assisted UKA had a learning curve of between six and 11 cases, compared with the Oxford mobile bearing UKA, which has a learning curve of 140 cases.^47-50^

In conclusion, our results demonstrate that patients undergoing R-UKA have a significantly lower all-cause revision rate and HLOS compared with those undergoing C-UKA. We found no difference in surgical duration, the odds of superficial surgical site infection, and PJI between the two groups. Our results suggest that R-UKA is the option of choice for managing symptomatic unicompartmental knee arthritis in carefully selected patients with isolated medial compartmental osteoarthritis, and particularly in the hands of lower-volume surgeons. Although we found that the HLOS in the R-UKA group was shorter, this should be interpreted with caution, given the non-contemporaneous nature of the two cohorts. The reduced revision risk justifies the continued use and development of R-UKA.

**Take home message**

- Robotic arm-assisted unicompartmental knee arthroplasty demonstrated a significantly lower risk of revision and a shorter hospital length of stay than conventional unicompartmental knee arthroplasty.

- Further research is warranted to determine the long-term clinical and economic implications of these findings.

## Data Availability

The datasets generated and analyzed in the current study are not publicly available due to data protection regulations. Access to data is limited to the researchers who have obtained permission for data processing. Further inquiries can be made to the corresponding author.

## References

[1] Jennings JM Kleeman-Forsthuber LT Bolognesi MP Medial unicompartmental arthroplasty of the knee J Am Acad Orthop Surg 2019 27 5 166 176 10.5435/JAAOS-D-17-00690 30407979

[2] Heyse TJ El-Zayat BF De Corte R et al. UKA closely preserves natural knee kinematics in vitro Knee Surg Sports Traumatol Arthrosc 2014 22 8 1902 1910 10.1007/s00167-013-2752-0 24213735

[3] Liddle AD Judge A Pandit H Murray DW Adverse outcomes after total and unicompartmental knee replacement in 101 330 matched patients: a study of data from the National Joint Registry for England and Wales Lancet 2014 384 9952 1437 1445 10.1016/S0140-6736(14)60419-0 25012116

[4] Liddle AD Pandit H Judge A Murray DW Patient-reported outcomes after total and unicompartmental knee arthroplasty Bone Joint J 2015 97-B 6 793 801 10.1302/0301-620X.97B6.35155 26033059

[5] Migliorini F Tingart M Niewiera M Rath B Eschweiler J Unicompartmental versus total knee arthroplasty for knee osteoarthritis Eur J Orthop Surg Traumatol 2019 29 4 947 955 10.1007/s00590-018-2358-9 30535643

[6] Albishi W AbuDujain NM Aldhahri M Alzeer M Unicompartmental knee replacement: controversies and technical considerations Arthroplasty 2024 6 1 21 10.1186/s42836-024-00242-6 38693586 PMC11064323

[7] D’Ambrosi R Milinkovic DD Migliorini F Mariani I Ursino N Hewett T Learning curve of persona partial knee (PPK) arthroplasty: a clinical trial BMC Musculoskelet Disord 2024 25 1 128 10.1186/s12891-024-07215-5 38341539 PMC10858461

[8] Zhang Q Zhang Q Guo W et al. The learning curve for minimally invasive Oxford phase 3 unicompartmental knee arthroplasty: cumulative summation test for learning curve (LC-CUSUM) J Orthop Surg Res 2014 9 81 10.1186/s13018-014-0081-8 25192976 PMC4173050

[9] Bunyoz KI Gromov K Troelsen A Starting up a lateral unicompartmental knee arthroplasty practice - is outcome affected? J Arthroplasty 2025 40 1 22 27 10.1016/j.arth.2024.07.005 39002768

[10] Badawy M Espehaug B Indrekvam K Havelin LI Furnes O Higher revision risk for unicompartmental knee arthroplasty in low-volume hospitals: data from 5,791 cases in the Norwegian Arthroplasty Register Acta Orthop 2014 85 4 342 347 10.3109/17453674.2014.920990 PMC4105763 24847789

[11] Abbas MJ Markel DC Hallstrom BR Zheng HT Charters MA The impact of surgeon volume on unicompartmental knee arthroplasty survivorship: a Michigan Arthroplasty Registry collaborative quality initiative database analysis J Arthroplasty 2025 40 1 15 21 10.1016/j.arth.2024.08.014 39147075

[12] Liddle AD Pandit H Judge A Murray DW Effect of surgical caseload on revision rate following total and unicompartmental knee replacement J Bone Joint Surg Am 2016 98-A 1 1 8 10.2106/JBJS.N.00487 26738897

[13] Lombardi AV Berend KR Berend ME et al. Current controversies in partial knee arthroplasty Instr Course Lect 2012 61 347 381 22301245

[14] Lewis P Gill D McAuliffe M et al. Hip, Knee and Shoulder Arthroplasty: 2024 Annual Report Adelaide Australian Orthopaedic Association National Joint Replacement Registry 2024 https://aoanjrr.sahmri.com/documents/10180/1798900/AOANJRR+2024+Annual+Report.pdf

[15] Ridgeway SR McAuley JP Ammeen DJ Engh GA The effect of alignment of the knee on the outcome of unicompartmental knee replacement J Bone Joint Surg Br 2002 84-B 3 351 355 10.1302/0301-620x.84b3.12046 12002491

[16] Barbadoro P Ensini A Leardini A et al. Tibial component alignment and risk of loosening in unicompartmental knee arthroplasty: a radiographic and radiostereometric study Knee Surg Sports Traumatol Arthrosc 2014 22 12 3157 3162 10.1007/s00167-014-3147-6 24972998

[17] Slaven SE Cody JP Sershon RA Ho H Hopper RH Fricka KB Alignment in medial fixed-bearing unicompartmental knee arthroplasty: the limb has a leg up on the component J Arthroplasty 2021 36 12 3883 3887 10.1016/j.arth.2021.08.015 34489145

[18] Ou D Ye Y Pan J et al. Finite element study of stress distribution in medial UKA under varied lower limb alignment Sci Rep 2024 14 1 25397 10.1038/s41598-024-74145-6 39455644 PMC11511828

[19] Marullo M Russo A Spreafico A Romagnoli S Mild valgus alignment after lateral unicompartmental knee arthroplasty led to lower functional results and survivorship at mean 8-year follow-up J Arthroplasty 2023 38 1 37 42 10.1016/j.arth.2022.07.009 35850420

[20] Liow MHL Tsai T-Y Dimitriou D Li G Kwon Y-M Does 3-dimensional in vivo component rotation affect clinical outcomes in unicompartmental knee arthroplasty? J Arthroplasty 2016 31 10 2167 2172 10.1016/j.arth.2016.03.003 27067168

[21] Kwon SC Jung HJ Lee JH Hyun JT Hwang JH Kim JI Robotic-assisted medial unicompartmental knee arthroplasty restored prearthritic alignment and led to superior functional outcomes compared with conventional techniques Knee Surg Sports Traumatol Arthrosc 2025 33 1 265 273 10.1002/ksa.12278 38796719

[22] Bell SW Anthony I Jones B MacLean A Rowe P Blyth M Improved accuracy of component positioning with robotic-assisted unicompartmental knee arthroplasty: data from a prospective, randomized controlled study J Bone Joint Surg Am 2016 98-A 8 627 635 10.2106/JBJS.15.00664 27098321

[23] St Mart J-P de Steiger RN Cuthbert A Donnelly W The three-year survivorship of robotically assisted versus non-robotically assisted unicompartmental knee arthroplasty Bone Joint J 2020 102-B 3 319 328 10.1302/0301-620X.102B3.BJJ-2019-0713.R1 32114810

[24] Lewis GD McAuliffe MJ Stoney JD et al. Knee and Shoulder Arthroplasty: 2025 Annual Report Adelaide Australian Orthopaedic Association National Joint Replacement Registry, AOA 2025 https://aoanjrr.sahmri.com/documents/d/guest/aoa-full-report-2025

[25] Kayani B Konan S Tahmassebi J Rowan FE Haddad FS An assessment of early functional rehabilitation and hospital discharge in conventional versus robotic-arm assisted unicompartmental knee arthroplasty Bone Joint J 2019 101-B 1 24 33 10.1302/0301-620X.101B1.BJJ-2018-0564.R2 30601042

[26] Banger M Doonan J Rowe P Jones B MacLean A Blyth MJB Robotic arm-assisted versus conventional medial unicompartmental knee arthroplasty: five-year clinical outcomes of a randomized controlled trial Bone Joint J 2021 103-B 6 1088 1095 10.1302/0301-620X.103B6.BJJ-2020-1355.R2 34058870 PMC8153511

[27] Bensa A Sangiorgio A Deabate L Illuminati A Pompa B Filardo G Robotic-assisted unicompartmental knee arthroplasty improves functional outcomes, complications, and revisions Bone Jt Open 2024 5 5 374 384 10.1302/2633-1462.55.BJO-2024-0030.R1 38690670 PMC11061807

[28] Agha RA Mathew G Rashid R Transparency in the reporting of Artificial INtelligence–the TITAN guideline Prem J Sci 2025 10 100082

[29] Vandenbroucke JP von Elm E Altman DG et al. Strengthening the Reporting of Observational Studies in Epidemiology (STROBE): explanation and elaboration Epidemiology 2007 18 6 805 835 10.1097/EDE.0b013e3181577511 18049195

[30] Kozinn SC Scott R Unicondylar knee arthroplasty J Bone Joint Surg Am 1989 71-A 1 145 150 10.2106/00004623-198971010-00023 2643607

[31] Saklad M Grading of patients for surgical procedures Anesthesiology 1941 2 3 281 284 10.1097/00000542-194105000-00004

[32] Charlson ME Pompei P Ales KL MacKenzie CR A new method of classifying prognostic comorbidity in longitudinal studies: development and validation J Chronic Dis 1987 40 5 373 383 10.1016/0021-9681(87)90171-8 3558716

[33] Kunze KN Farivar D Premkumar A Cross MB Della Valle AG Pearle AD Comparing clinical and radiographic outcomes of robotic-assisted, computer-navigated and conventional unicompartmental knee arthroplasty: a network meta-analysis of randomized controlled trials J Orthop 2021 25 212 219 10.1016/j.jor.2021.05.012 34045825 PMC8144096

[34] Gilmour A MacLean AD Rowe PJ et al. Robotic-arm-assisted vs conventional unicompartmental knee arthroplasty. the 2-year clinical outcomes of a randomized controlled trial J Arthroplasty 2018 33 7S S109 S115 10.1016/j.arth.2018.02.050 29627257

[35] Cobb J Henckel J Gomes P et al. Hands-on robotic unicompartmental knee replacement J Bone Joint Surg Br 2006 88-B 2 188 197 10.1302/0301-620X.88B2.17220 16434522

[36] Karia M Masjedi M Andrews B Jaffry Z Cobb J Robotic assistance enables inexperienced surgeons to perform unicompartmental knee arthroplasties on dry bone models with accuracy superior to conventional methods Adv Orthop 2013 2013 1 481039 10.1155/2013/481039 23862069 PMC3703799

[37] Matassi F Innocenti M Giabbani N et al. Robotic-assisted unicompartmental knee arthroplasty reduces components’ positioning differences among high- and low-volume surgeons J Knee Surg 2022 35 14 1549 1555 10.1055/s-0041-1727115 33853154

[38] Fontalis A Raj RD Haddad IC et al. Length of stay and discharge dispositions following robotic arm-assisted total knee arthroplasty and unicompartmental knee arthroplasty versus conventional technique and predictors of delayed discharge Bone Jt Open 2023 4 10 791 800 10.1302/2633-1462.410.BJO-2023-0126.R1 37852620 PMC10614696

[39] Shearman AD Sephton BM Wilson J Nathwani DK Robotic-assisted unicompartmental knee arthroplasty is associated with earlier discharge from physiotherapy and reduced length-of-stay compared to conventional navigated techniques Arch Orthop Trauma Surg 2021 141 12 2147 2153 10.1007/s00402-021-04207-1 34652516

[40] Xu J-Z Li L-L Fu J et al. Comparison of serum inflammatory indicators and radiographic results in MAKO robotic-assisted versus conventional total knee arthroplasty for knee osteoarthritis: a retrospective study of Chinese patients BMC Musculoskelet Disord 2022 23 1 418 10.1186/s12891-022-05373-y 35509075 PMC9066791

[41] Goh GS Haffar A Tarabichi S Courtney PM Krueger CA Lonner JH Robotic-assisted versus manual unicompartmental knee arthroplasty: a time-driven activity-based cost analysis J Arthroplasty 2022 37 6 1023 1028 10.1016/j.arth.2022.02.029 35172186

[42] Varughese I Whitehouse SL Donnelly WJ Crawford RW The cost effectiveness of unicompartmental versus total knee arthroplasty J Knee Surg 2024 37 1 66 72 10.1055/a-1967-2346 36283414

[43] Speckman RA Summary measures and measures of effect: summarizing and comparing outcomes in rehabilitation research. Part 3: time‐to‐event outcomes, unequal follow‐up times, and survival analysis PM R 2021 13 2 204 210 10.1002/pmrj.12535 33314788

[44] Abu Al-Rub Z Lamb JN West RM Yang X Hu Y Pandit HG Survivorship of fixed vs mobile bearing unicompartmental knee replacement: a systematic review and meta-analysis of sixty-four studies and National Joint Registries Knee 2020 27 5 1635 1644 10.1016/j.knee.2020.09.004 33010783

[45] Tay ML Bolam SM Maxwell AR Hooper GJ Monk AP Young SW Similar survivorship but different revision reasons for uncemented mobile-bearing and cemented fixed-bearing medial UKA: a long-term population-based cohort study of 2,015 patients J Bone Joint Surg Am 2023 105-A 10 10.2106/JBJS.22.00686 36812351

[46] Crizer MP Haffar A Battenberg A McGrath M Sutton R Lonner JH Robotic assistance in unicompartmental knee arthroplasty results in superior early functional recovery and is more likely to meet patient expectations Adv Orthop 2021 2021 1 4770960 10.1155/2021/4770960 34336292 PMC8298171

[47] Bush AN Ziemba-Davis M Deckard ER Meneghini RM An experienced surgeon can meet or exceed robotic accuracy in manual unicompartmental knee arthroplasty J Bone Joint Surg Am 2019 101-A 16 1479 1484 10.2106/JBJS.18.00906 31436656

[48] Tay ML Carter M Bolam SM Zeng N Young SW Robotic-arm assisted unicompartmental knee arthroplasty system has a learning curve of 11 cases and increased operating time Knee Surg Sports Traumatol Arthrosc 2023 31 3 793 802 10.1007/s00167-021-06814-2 34981161

[49] Kayani B Konan S Pietrzak JRT Huq SS Tahmassebi J Haddad FS The learning curve associated with robotic-arm assisted unicompartmental knee arthroplasty Bone Joint J 2018 100-B 8 1033 1042 10.1302/0301-620X.100B8.BJJ-2018-0040.R1 30062950

[50] Morikawa LH Combs DB Andrews SN Mathews K Nakasone CK Component positioning of the first 300 mobile bearing unicompartmental knee arthroplasties J Orthop 2021 27 9 12 10.1016/j.jor.2021.08.002 34413583 PMC8363760

